# Examination of Transmission Zeros in the MIMO Sensor-Based Propagation Environment Using a New Geometric Procedure

**DOI:** 10.3390/s24030954

**Published:** 2024-02-01

**Authors:** Dariusz Pączko, Wojciech P. Hunek

**Affiliations:** 1 Department of Mathematics and IT Applications, Opole University of Technology, 45-758 Opole, Poland; d.paczko@po.edu.pl; 2 Department of Control Science and Engineering, Opole University of Technology, Prószkowska 76, 45-758 Opole, Poland

**Keywords:** transmission zeros, MIMO telecommunications systems, geometric approach, Smith–McMillan decomposition, computational burden

## Abstract

In this paper, we propose the application of a new geometric procedure in order to calculate a set of transmission zeros of a propagation environment. Since the transmission zeros play a crucial role in modern communication systems, there is a need to apply the efficient solutions characterized by a maximum speed operation. It turns out that the classical method based on the Smith–McMillan factorization is time-consuming, so its contribution to the detection of transmission zeros could be unsatisfactory. Therefore, in order to fill the gap, we present a new algorithm strictly dedicated to the multivariable telecommunications systems described by the transfer-function approach. Consequently, a set of new achievements resulted, particularly in terms of computational efforts. Indeed, the proposed procedure allows us to overcome obstacles derived from technological limitations. The representative simulation examples confirm the great potential of this new method. Finally, it has been pointed out that the newly introduced geometric-originated approach has significantly reduced the computational burden. Indeed, for the randomly selected matrix of the 5×5 dimension describing the sensor-related propagation environment, two representative scenarios were performed in order to manifest the crucial properties. In the first scenario, the sets of multiple transmission zeros were analyzed, ultimately leading to intriguing results. The Smith–McMillan solution took three times longer to discover the mentioned sets. On the other hand, the second instance brought us the same result. Naturally, the discussed difference has increased as a function of the number of matrix elements. For the square matrices involving 100 components, we have observed the respective differences, both over $Q_{I}=100$ and $Q_{II}=60$. It should be emphasized that the finding derived from the Smith–McMillan factorization corresponds to the geometric-related approach in the context of some mechanisms. This is particularly visible when appointing the greatest common divisors.

## 1. Introduction

The phenomenon of transmission zeros remains significant in the modern age of science and engineering [1,2,3,4,5,6,7]. They can be found in several theoretical and practical applications, such as signal processing and control theory branches [8]. Solid illustrations certifying the contribution of transmission zeros to the massive MIMO and mmWave technologies are expressed in Refs. [9,10,11]. It should be emphasized that a comprehensive overview of zeros of multivariable systems, including the discussed transmission zeros, along with the relationships observed between them, is addressed in Ref. [12]. Since the transmission zeros are crucial during the identification of the dynamic properties of systems in both the time and frequency domains, there is a need to apply a set of efficient tools that allow us to operate in real-time conditions [13,14]. This is important due to the fact that such an operation identifies the transmission zeros that are strictly responsible for blocking the input signal runs [15]. Indeed, existing approaches devoted to the calculation of transmission zeros even perform well, but they may be ineffective in the case of complex plants. In such a scenario, the large matrices describing behaviors originating from the transfer function may be inaccurately expressed due to the high computational effort. Apparently, the most important method encompassing the Smith–McMillan factorization enables a determination of discussed zeros in a reasonable time, which could be shortened for more demanding applications [12]. For this purpose, a new geometric-oriented algorithm was recently introduced and its valuable properties were presented to the world scientific society [16]. Henceforth, stable and unstable transmission zeros can be calculated faster in comparison to the broadly accepted Smith–McMillan-based approach in order to meet the predefined time regime in a wide range of well-established real-life implementations. Clearly, the reduced number of mathematical iterations, combined with the decreased computational burden and time consumption, presents a major advantage of the original solution. In the end, the existence of transmission zeros can straightforwardly result in the malfunction of the entire system.

It should also be emphasized that the geometric method can be applied to calculate the so-called control zeros [1,8]. Since the set of control zeros covers the set of transmission zeros for square systems, there is the possibility of obtaining a reasonable tool. Note that control zeros extend the transmission ones to non-square plants and characterize the stable potential of the inverse model control design. In fact, stable control zeros offer stable deterministic perfect control, and the stochastic version is referred to as minimum variance control. On the other hand, a set of unstable transmission zeros provides unstable control laws. The first instance advocates for minimum-phase systems, whereas the second one is closely associated with non-minimum-phase objects. The phenomenon of control zeros still attracts interest in the employment of various generalized inverses of non-square matrices.

Taking into account the above considerations, the main contributions of the manuscript are outlined as follows:

1. We effectively present the new geometric method addressing the calculation of transmission zeros.
2. The discussed method is strictly associated with the multivariable systems encompassing different numbers of input and output variables.
3. The approach undeniably outperforms the classical solution based on the Smith–McMillan decomposition.
4. The advantages of the proposed method are understood in terms of reducing the computational effort, ultimately leading to the adaptation of the algorithm to real-time operations.
5. A detrimental effect of stable and unstable transmission zeros is eliminated as a result of the running procedure. Henceforth, the innovative tool can also be used for systems without discussed zeros.
6. Since the geometric-originated strategy can be used in various domains, it provides a solid background for other theoretical and practical applications.
7. The contribution of the new geometric method to a wide range of physical scenarios is clearly visible, e.g., it can be used in advanced signal processing as well as in modern control theory and practice.
8. The great potential of the newly introduced approach provides a set of open problems. For example, the method can be evaluated with respect to its applicability to ill-conditioned matrices.

This paper is organized in the following manner: Section 2 describes the non-square systems in the form of a propagation environment. The classical method allowing a calculation of transmission zeros based on the Smith–McMillan decomposition is presented in Section 3. Section 4 shows a recently introduced, newly efficient method associated with the geometric approach. The comparative studies encompassing the two discussed algorithms are also presented in this section. Several scenarios that refer to different sets of transmission zeros have resulted in constructive feedback, which, along with the open problems, are formulated in the last section of the manuscript.

## 2. System Description

One of the basic objects describing systems with multiple inputs and outputs is the transfer function matrix G(s), where the number of rows, $n_{y}$, corresponds to the number of outputs and the number of columns, $n_{u}$, is equal to the number of inputs. Matrix G(s) is, therefore, of the following form:

(1) $$G\left(s\right)=\left[\begin{array}{cccc} g_{1,1}\left(s\right) & g_{1,2}\left(s\right) & \ldots & g_{1,n_{u}}\left(s\right)\\ g_{2,1}\left(s\right) & g_{2,2}\left(s\right) & \ldots & g_{2,n_{u}}\left(s\right)\\ \vdots & \vdots & \ddots & \vdots \\ g_{n_{y},1}\left(s\right) & g_{n_{y},2}\left(s\right) & \ldots & g_{n_{y},n_{u}}\left(s\right) \end{array}\right],$$

where entries $g_{i,j}\left(s\right)$, $i=1,\ldots ,n_{y}$, $j=1,\ldots ,n_{u}$ denote rational functions $g_{i,j}\left(s\right)=\frac{L_{i,j}\left(s\right)}{M_{i,j}\left(s\right)}$ of complex variable *s* with polynomials $L_{i,j}\left(s\right)$ and $M_{i,j}\left(s\right)$ of appropriate degrees. Usually, the normal rank of this matrix is denoted by *r*, with $r\leq \min(n_{u},n_{y})$. However, when transmission zeros occur, this matrix loses its rank. Simply examining the zeros of the individual elements of the matrix, unfortunately, does not contribute to the determination of transmission zeros. One of several solutions to this problem is the Smith–McMillan factorization. Although the algorithm of this method is somewhat complicated and complex, it allows us to effectively determine the transmission zeros of the transfer function matrix. Another approach to determining the transmission zeros is the geometric method, based on certain properties of the rank of the product of matrix G(s) and its Hermitian form $G^{H}\left(s\right)$. It allows us to determine the transmission zeros and their multiplicity in a more intuitive way, and most importantly, in a shorter computational time.

**Figure 1:**
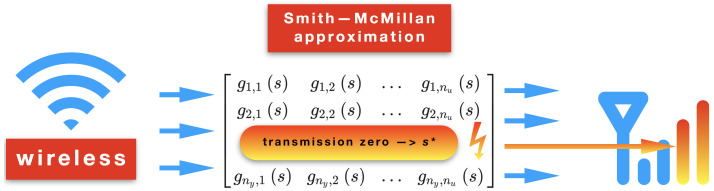
The transmission-blocking of a signal process in the propagation environment.

Since transmission zeros play an important role in the study of regulation, structural stability, decoupling, and servo design, and their current state-of-the-art is now well-established, their contribution related to the propagation environment in the design of telecommunication systems, described by matrix G(s) of Equation (1), can be seen in Figure 1. In short, transmission zeros in this context are specific complex frequencies in which signal transmission through the system is blocked.

Having clarified the basics of the notation and the resulting problems, in the next section, we will focus on a detailed examination of the concept of transmission zeros.

## 3. Smith–McMillan Factorization for Multivariable Systems

Based on elementary transformations, the transfer function matrix G(s) can be brought to the Smith–McMillan form M(s), preserving the rank equivalence of the two matrices. There is also a direct algorithm for determining M(s) based on calculating the successive largest common divisors of the corresponding minors of a given degree (see [17]). This method, although tedious, makes it possible to find a matrix M(s) in the following form:

(2) $$M\left(s\right)=\left[\begin{array}{cccccc} \frac{\epsilon_{1}\left(s\right)}{\psi_{1}\left(s\right)} & 0_{1,2} & \ldots & 0_{1,n_{y}} & \ldots & 0_{1,n_{u}}\\ 0_{2,1} & \frac{\epsilon_{2}\left(s\right)}{\psi_{2}\left(s\right)} & \ldots & 0_{2,n_{y}} & \ldots & 0_{2,n_{u}}\\ \vdots & \vdots & \ddots & \vdots & \ddots & \vdots \\ 0_{n_{y},1} & 0_{n_{y},2} & \ldots & \frac{\epsilon_{n_{y}}\left(s\right)}{\psi_{n_{y}}\left(s\right)} & \ldots & 0_{n_{y},n_{u}} \end{array}\right],$$

where polynomials $\epsilon_{i}\left(s\right)$ and $\psi_{i}\left(s\right)$ are co-prime.

According to the rank equality of matrices M(s) and G(s), it is easy to determine the transmission zeros of G(s) as the roots of the polynomial:

(3) $$z\left(s\right)=\epsilon_{1}\left(s\right)\epsilon_{2}\left(s\right)\cdots \epsilon_{n_{y}}\left(s\right),$$

and thus the poles as the roots of the function

(4) $$p\left(s\right)=\psi_{i}\left(s\right)\psi_{2}\left(s\right)\cdots \psi_{n_{y}}\left(s\right).$$

Of course, this definition of transmission zeros is inherent to the Smith–McMillan method. Therefore, throughout the remainder of this paper, we will use a more intuitive definition based on the loss of the matrix rank, which does not contradict the definition based on the roots of the polynomial as in Equation (3), and is closely related to it.

**Definition** **1.** *Let G(s) be an $n_{y}\times n_{u}$-dimensional transfer function matrix defined for s∈X⊂C and the normal rank of G(s) is equal to $r\leq \min(n_{y},n_{u})$, i.e., rankG(s)=r for almost all values of s.*

The transmission zeros of G(s) are called values of $s^{*}\in X$ for which matrix G(s) loses its rank; that is,

$$\mathrm{rank}G\left(s^{*}\right)<\max_{s\in X}\mathrm{rank}G\left(s\right)\leq \min\left(n_{y},n_{u}\right)\;for\;all\;s\neq s^{*}.$$

**Remark** **1** ([12])**.** *In the literature, it is common to find an alternative definition of transmissive zeros. In this definition, the Rosenbrok matrix in the form of*

$$R\left(s\right)=\left[\begin{array}{cc} sI_{n}-A & B\\ -C & D \end{array}\right],$$

*loses its rank, where matrices A,B,C, and D represent the linear state-space system. It has been shown that*

rankR(s)=n+rankG(s).

*However, it should be noted that the rank of matrix R(s) can decrease for points that are both eigenvalues of matrix A and poles of matrix G(s). Therefore, in our definition of transmission zeros, we exclude these points since matrix G(s) is not defined at those points due to pole-zero cancellation.*

## 4. A New Method of Calculation of Transmission Zeros

A new geometric method has been proposed to compute transmission zeros based on the equality of the rank of the matrix A(s) depending on the complex variable *s* and the rank of the product of matrix $A^{H}\left(s\right)A\left(s\right)$ or $A\left(s\right)A^{H}\left(s\right)$, where $A^{H}\left(s\right)$ denotes the Hermitian conjugate of the matrix A(s). This method is less tedious than the Smith–McMillan procedure. The property is formulated as the following lemma.

**Lemma** **1** (see [16])**.** *Let a matrix A(s) with dimensions m×n be defined for s∈X⊂C. Then, the following equalities are satisfied*

(5) $$\begin{array}{cc} \mathrm{rank}A(s) & =\mathrm{rank}\left(A^{H}\left(s\right)A\left(s\right)\right)=\mathrm{rank}(A\left(s\right)A^{H}\left(s\right)), \end{array}$$

*for all values of parameter s∈X.*

Lemma 1 states that transfer matrix G(s) satisfies the condition

$$\mathrm{rank}G\left(s\right)=\mathrm{rank}\left(G\left(s\right)G^{H}\left(s\right)\right)=\mathrm{rank}\left(G^{H}\left(s\right)G\left(s\right)\right).$$

This implies that the procedure for determining transmission zeros for rectangular transfer matrix G(s) can be replaced by the corresponding procedure for square matrices $G\left(s\right)G^{H}\left(s\right)$ or $G^{H}\left(s\right)G\left(s\right)$, depending on whether $n_{y}$ is smaller or larger than $n_{u}$. If $n_{y}=n_{u}$, any of the given products can be chosen for the calculation.

The proposed method involves more simplifications than just replacing the rectangular matrix with a square one. Matrices $G\left(s\right)G^{H}\left(s\right)$ and $G^{H}\left(s\right)G\left(s\right)$ are positive semi-definite, having only real non-negative eigenvalues. The number of positive eigenvalues corresponds to the rank of the respective product of matrices $G\left(s\right)G^{H}\left(s\right)$ or $G^{H}\left(s\right)G\left(s\right)$. The product of all eigenvalues is equal to the determinant of the corresponding product of $G\left(s\right)G^{H}\left(s\right)$ or $G^{H}\left(s\right)G\left(s\right)$. If the determinant for any s∈C is not zero, then we can conclude that the product of matrices $G\left(s\right)G^{H}\left(s\right)$ or $G^{H}\left(s\right)G\left(s\right)$ does not have a transmission zero. This means that transfer matrix G(s) also does not have a transmission zero. The reason for this is that matrix $G\left(s\right)G^{H}\left(s\right)$ or $G^{H}\left(s\right)G\left(s\right)$ is positive definite, which means it only has positive eigenvalues. The number of these eigenvalues is equal to the rank of the matrix.

In the simplest case, analyzing the transmission zeros of the transfer matrix G(s) involves solving equations, such as

$$\det\left(G\left(s\right)G^{H}\left(s\right)\right)=0\;\mathrm{for}\;n_{y}\leq n_{u},$$

or

$$\det\left(G^{H}\left(s\right)G\left(s\right)\right)=0\;\mathrm{for}\;n_{y}\geq n_{u}.$$

However, if the corresponding determinant is identically equal to zero for any s∈C, we search for elements among all sub-determinants (leading minors) of a lower degree than $n_{y}$ or $n_{u}$, respectively. This is based on the Cauchy interlacing theorem.

**Theorem** **1** (Cauchy interlacing theorem)**.** *Let A be an n×n symmetric matrix and let B be a principal submatrix of A of order m<n, obtained by deleting both rows and columns with the same index number. If $\lambda_{1}\leq \ldots \leq \lambda_{n}$ are the eigenvalues of matrix A and $\beta_{1}\leq \ldots \beta_{m}$ are the eigenvalues of matrix B, then*

$$\lambda_{k}\leq \beta_{k}\leq \lambda_{k+n-m}\;\mathrm{for}\;k=1,\ldots ,m,$$

*and if m=n−1, then*

$$\lambda_{1}\leq \beta_{1}\leq \lambda_{2}\leq \beta_{2}\leq \ldots \beta_{n-1}\leq \lambda_{n}.$$

The presented properties and theorems enable us to formulate a condition for determining the transmission zeros of the transfer matrix G(s). This is based on the product of $G\left(s\right)G^{H}\left(s\right)$ or $G^{H}\left(s\right)G\left(s\right)$.

**Theorem** **2.** A transfer-function matrix G(s) of dimensions $n_{y}\times n_{u}$ with normal rank $r\leq \min(n_{y},n_{u})$ is defined for s∈X⊂C. Matrix G(s) has a transmission zero at point $s^{*}\in X$ iff all the r×r principal minors of $G^{H}\left(s\right)G\left(s\right)$ or $G\left(s\right)G^{H}\left(s\right)$ are simultaneously equal to zero at this point, i.e., the following equalities occur

(6) $${\left[G^{H}\left(s^{*}\right)G\left(s^{*}\right)\right]}_{J,J}=0\;\forall J\subseteq \left\{1,2,\ldots ,n_{u}\right\},$$

*or*

(7) $${\left[G\left(s^{*}\right)G^{H}\left(s^{*}\right)\right]}_{K,K}=0\;\forall K\subseteq \left\{1,2,\ldots ,n_{y}\right\},$$

*where subscript J,K indicates that the minor of the corresponding matrix product is calculated, and its rows, r, have indices in set J, and r columns have indices in set K.*

### 4.1. Computational Effort

When comparing the computational complexity of determining the transmission zeros between the two methods, it is important to note that the new algorithm requires fewer calculated principal minors than the Smith–McMillan method, which calculates all minors. In the first case, for a matrix A with dimensions m×n, we only need to determine min(m,n)r prime minors of degree *r*. However, in the Smith–McMillan method, this number increases to ∑k=1min(m,n)mk·nk. For m=n, the asymptotic notation shows that we have $O(2^{n})$ and $O(2^{2n})$ for the two cases, respectively.

However, it should be taken into account that the new method is based on the product of the corresponding transfer matrices. As a result, the related principal minors, although fewer in number, will be more complex, which may affect the performance of the new method. Additionally, solving the more complicated system of Equations (6) or (7) will be a burden. Although the conditions of Theorem 2 provide an alternative to the existing Smith–McMillan method, they may not be sufficient to overcome real-world obstacles.

To enable the practical application of the new method, we will employ the Cauchy–Binnet formula for the minor product of matrices, so that for (6), we have

(8) $$\begin{array}{c} {\left[G^{H}\left(s\right)G\left(s\right)\right]}_{J,J}=\sum_{K}{\left[G^{H}\left(s\right)\right]}_{J,K}{\left[G\left(s\right)\right]}_{K,J}=\sum_{K}{\left[\bar{G(s)}\right]}_{K,J}{\left[G\left(s\right)\right]}_{K,J}=\sum_{K}{\left|{\left[G\left(s\right)\right]}_{K,J}\right|}^{2}, \end{array}$$

where the summation proceeds over all *r* element subsets of $K\subseteq \{1,2,\ldots ,n_{y}\}$. Condition (6) can be replaced by the following condition:

(9) $$\sum_{K}{\left|{\left[G\left(s\right)\right]}_{K,J}\right|}^{2}=0.$$

This means that all the minors ${\left[G\left(s\right)\right]}_{K,J}$ must simultaneously be zero at the same point *s* for all *r*-element sets *K*. Therefore, the condition satisfies the greatest common divisor of the corresponding minors, i.e.,

(10) $$GCD({\left[G\left(s\right)\right]}_{K,J})=0.$$

Similarly, by transforming Equation (7), we obtain the condition $GCD({\left[G\left(s\right)\right]}_{J,K})=0$.

Both methods involve the greatest common divisor of corresponding minors. However, the new solution only calculates those of degree *r*, while the Smith–McMillan method evaluates all minors from degree 1 to *r*. Additionally, if the minor ${\left[G\left(s\right)\right]}_{K,J}$ is non-zero for certain sets of row and column indices, *K* and *J*, there is no need to compute the remaining minors of the corresponding degree, *r*. If the determinant of matrix $G^{H}\left(s\right)G\left(s\right)$ or $G\left(s\right)G^{H}\left(s\right)$ is not equal to zero, meaning that the only principal minors of degrees $n_{u}$ and $n_{y}$ are not equal to zero, there is no need to compute the remaining principal minors of lower degrees. This is because the corresponding product of matrix $G^{H}\left(s\right)G\left(s\right)$ or $G\left(s\right)G^{H}\left(s\right)$ is already positive definite, and the transfer function matrix G(s) has no transmission zero.

**Figure 2:**
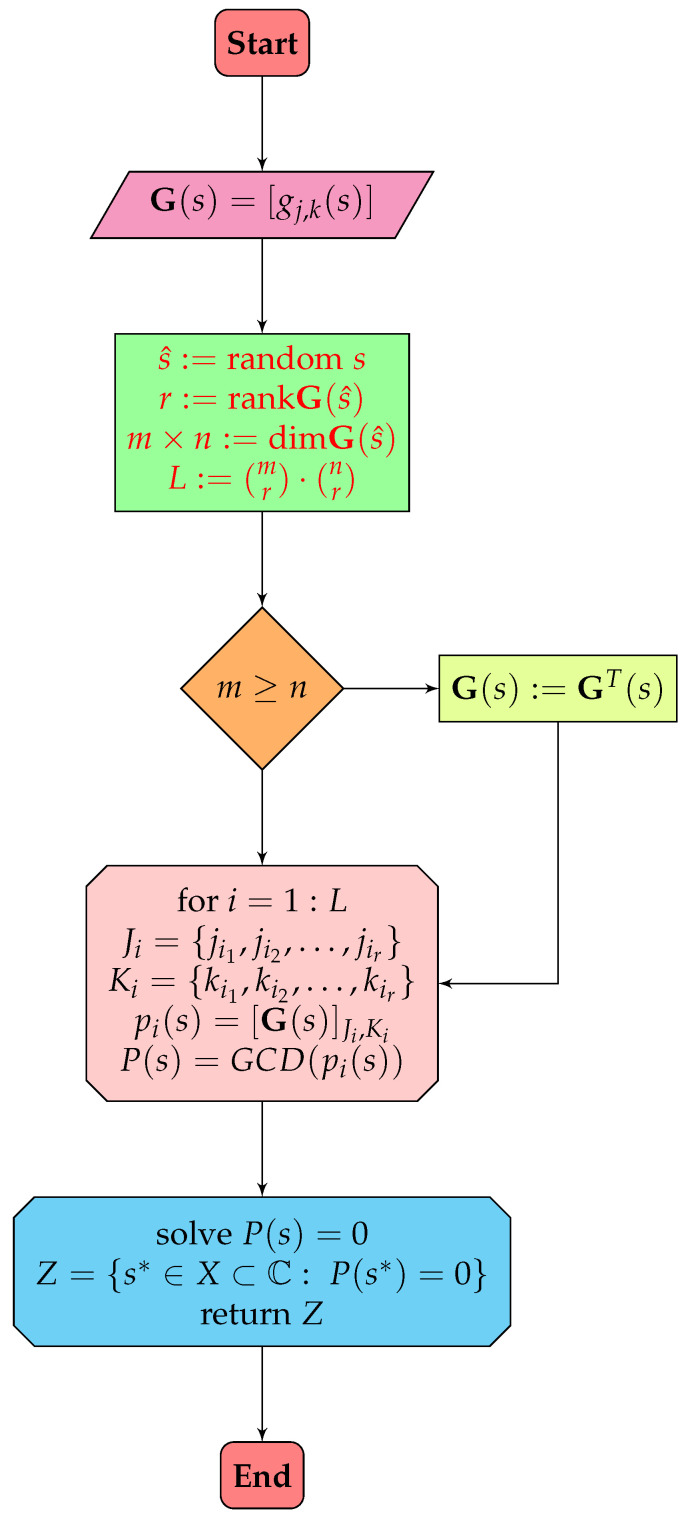
A scheme of the calculation of transmission zeros in the new geometric method.

Figure 2 summarizes the properties described, depicting the operation of the algorithm for the new geometric method used to identify transmission zeros. On the other hand, Algorithm 1 shows the pseudocode of the new procedure.

**Algorithm 1** Geometric method algorithm: The pseudocode of the geometric method procedure.
**function** GMA(G(s))
  {m,n} ← dimensions of G(s)  d(s) ← the least common multiple of the denominators of all elements in G(s)  P(s) ← d(s)G(s)  *r* ← the rank of $G(\hat{s})$ for random $\hat{s}$.  g(s) ← the greatest common divisor of all the r×r minors of P(s)  $s_{j}$ ← roots of g(s)=0  **return** $s_{j}$                     ▹ transmission zeros of G(s)
**end function**



### 4.2. Algorithm Running Time

**Figure 3:**
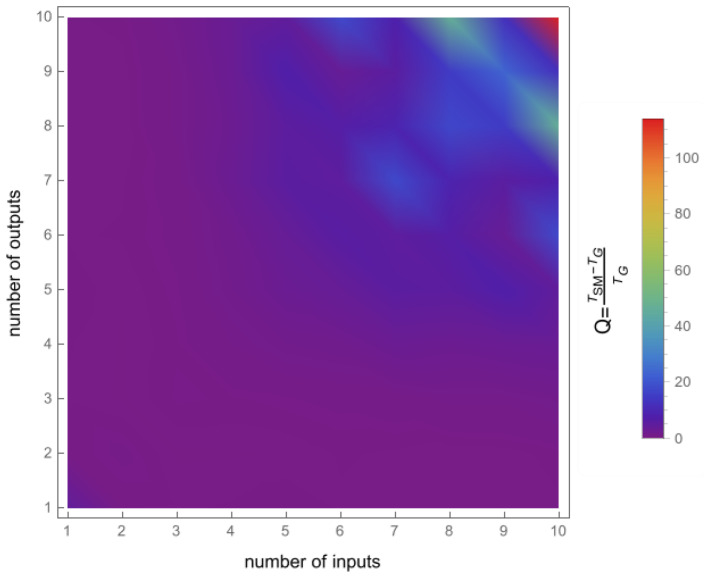
The quotient $Q=\frac{T_{\mathrm{SM}}-T_{G}}{T_{G}}$, where $T_{\mathrm{SM}}$ is the execution time for the Smith–McMillan approach and $T_{G}$ is the execution time in the new geometric method. The case with transmission zeros in the transfer function matrix G(s).

**Figure 4:**
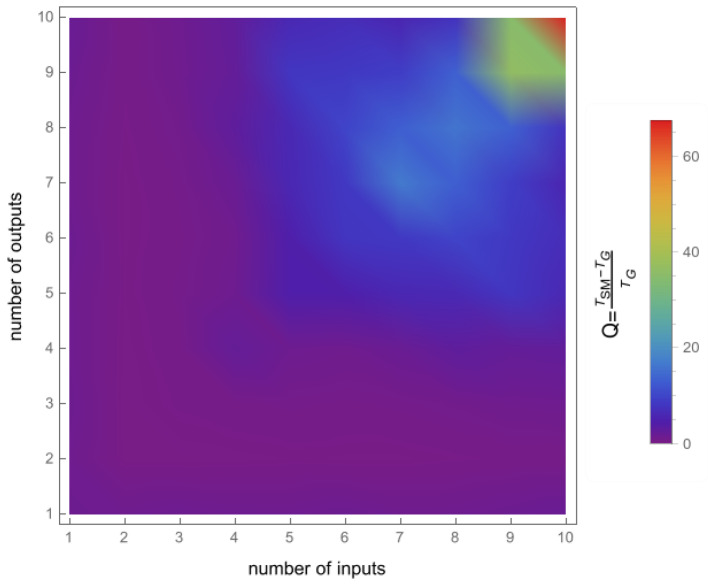
The quotient $Q=\frac{T_{\mathrm{SM}}-T_{G}}{T_{G}}$, where $T_{\mathrm{SM}}$ is the execution time for the Smith–McMillan approach and $T_{G}$ is the execution time in the new geometric method. The case with no transmission zeros in the transfer function matrix G(s).

To confirm the effectiveness of the new geometric method, we compared its running time to that of the Smith–McMillan approach. We performed a corresponding procedure for both algorithms in the Mathematica environment and tested matrices G(s)=UA(s)V, ranging from 1×1 to 10×10. Matrices U and V are random unimodular matrices, while $A\left(s\right)={\left[a_{ij}\left(s\right)\right]}_{m\times n}$ is a matrix in which the elements $a_{ij}=0$ for i≠j and $a_{ii}$ are random rational functions. The first set of tests involved randomly selected stable transmission zeros of matrix G(s) as elements of the functions $a_{ii}$. In the second set of tests, it was assumed that $a_{ii}$ had no roots. The results of both tests are shown in Figure 3 and Figure 4, clearly demonstrating the superiority of the new geometric method over the Smith–McMillan algorithm. This superiority is particularly evident as the matrix dimension increased, with a running time that was more than 100 times faster for a 10×10 matrix.

### 4.3. Discussions of the Obtained Results

**Figure 5:**
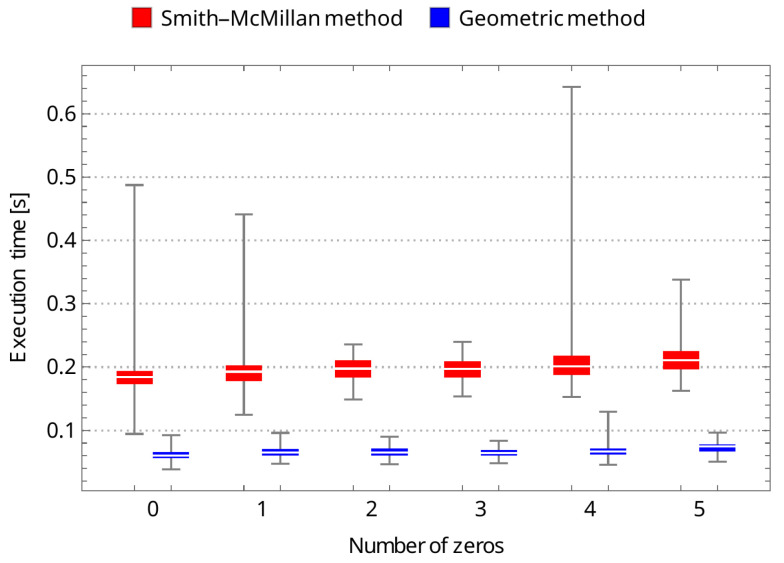
The boxplot-based representation of execution time tests related to Table 1.

**Figure 6:**
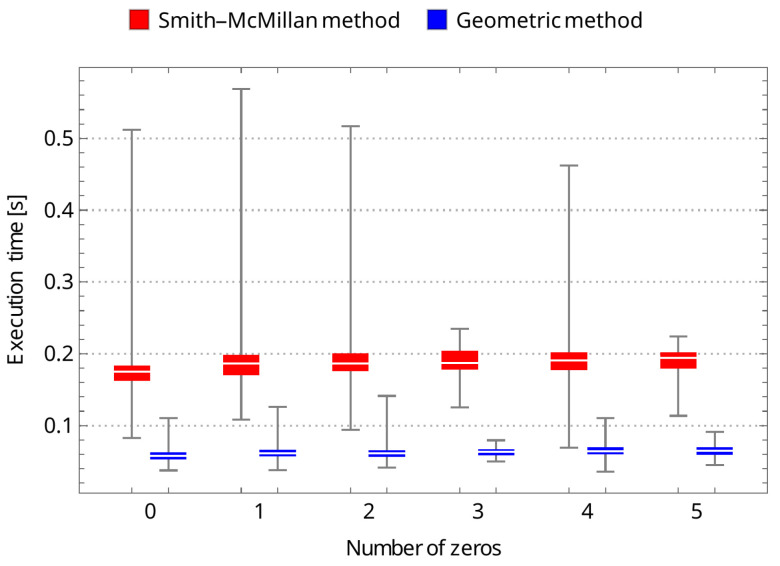
The boxplot-based representation of execution time tests related to Table 2.

As a supplement to the conducted studies, an additional set of tests is proposed throughout this section. Following the crucial statements of the previous unit, the new geometric approach turned out to be more efficient than a solution strictly associated with the Smith–McMillan decomposition. All simulation examples covering a randomly selected matrix of dimensions 5×5 have also confirmed the superiority of the former method over the classical one. The predefined cases encompassing multiple zeros clearly advocate for the geometric-originated approach in the context of computational consumption (see Table 1). The same can be observed under different transmission zero instances as shown in Table 2. In addition, statistical representations of Table 1 and Table 2 are depicted in Figure 5 and Figure 6. From the figures, it can be clearly seen that—regardless of the number of transmission zeros or their multiplicity—the median execution time for the geometric method is about three times less than that of the Smith–McMillan method in each case studied. It can also be noted that the execution time is not affected by the multiplicity of transmission zeros; in both cases, the time is at a similar level. However, the time is slightly shorter when there are no zeros. It should finally be noted that the simulation studies were carried out using a personal computer: Dell, Inc., Inspiron 7590, supported by Intel Core^™^ i7-9750H x 12, with 16.0 GB RAM, and NVIDIA GeForce GTX 1650.

## 5. Conclusions and Open Problems

Throughout this paper, we presented an efficient algorithm strictly dedicated to the calculation of transmission zeros. Special attention was placed on a computational effort, which was significantly reduced with regard to the classical method employing the Smith–McMillan approach. Apparently, the geometric-oriented procedure turned out to be less time-consuming when applied to every telecommunications system encompassing several specified sensors. This was confirmed by the asymptotic computational complexity as well as by numerous tests. Since the new method has brought visible benefits, numerous open problems have arisen. The first important issue is related to the application of the new methodology to real-life measurement systems. An extension of the discussed procedure to the ill-conditioned plants seems to be a second problem worthy of extensive research studies in the near future.

## Figures and Tables

**Table 1:** The Smith–McMillan method vs. geometric approach: the multiple transmission zeros instances—medians of execution times, depending on the number of transmission zeros (for $N_{i}=100$ samples, i=0,…,5).

Number of Trans. Zeros (5×5-Matrix)	0	1	2	3	4	5
Smith–McMillan decomposition (s)	0.184565	0.192738	0.197581	0.197219	0.201427	0.210699
geometric approach (s)	0.0600365	0.065302	0.064841	0.0645565	0.066624	0.074523

**Table 2:** The Smith–McMillan method vs. geometric approach: the different transmission zeros instances—medians of execution times, depending on the number of transmission zeros (for $N_{i}=100$ samples, i=0,…,5).

Number of Trans. Zeros (5×5-Matrix)	0	1	2	3	4	5
Smith–McMillan decomposition (s)	0.174914	0.186208	0.186246	0.187248	0.190912	0.19419
geometric approach (s)	0.0577405	0.061239	0.0614765	0.063528	0.063922	0.065036

## Data Availability

Data are contained within the article.
